# Single-cell multi-omics dissection of RevitalAge Markers uncovers age-dependent immunotherapy resistance and druggable targets in melanoma

**DOI:** 10.1186/s13062-026-00860-x

**Published:** 2026-06-16

**Authors:** Hanxiao Zhou, Benliang Wei, Wenlu Tan, Ji Shi, Changyuan Ren, Jinhao Zhang, Changlin Yang, Zheng Zhao, Shangwei Ning

**Affiliations:** 1 Beijing Neurosurgical Institute, Capital Medical University, Beijing, 100070 China; 2 College of Bioinformatics Science and Technology, Harbin Medical University, Harbin, Heilongjiang 150081 China; 3 Beijing Tiantan Hospital, Capital Medical University, Beijing, 100070 China; 4 Department of Central Laboratory, Beijing Obstetrics and Gynecology Hospital, Capital Medical University, Beijing Maternal and Child Health Care Hospital, Beijing, 100026 China

**Keywords:** Multi-omics, Machine learning, Therapeutic sensitivity, Aging

## Abstract

Aging-related molecular reprogramming profoundly influences melanoma progression and therapeutic sensitivity, yet underlying mechanisms remain poorly understood. We constructed a comprehensive multi-omics atlas integrating mutations, transcription, methylation, and copy number variations, utilizing the MOFA algorithm to uncover key age-driving factors. Among 20 factors, Factor6 exhibited significant negative age correlation and survival protection (HR = 0.95, *P* = 4.494e-05), defined as “RevitalAge Marker (RAM)”. Integration of three single-cell RNA sequencing datasets revealed 4,796 RAM+ cells (predominantly endothelial cells and fibroblasts) exerting tumor-suppressive functions through VEGFA-VEGFR2 angiogenic pathways and mitochondrial ATP synthesis, while 7,229 RAM- cells (dominated by malignant cells) exhibited enhanced EMT, hypoxic adaptation, and MAPK signaling activation. RAM+ cells were governed by ZFP42 and IRF8 maintaining anti-tumor immunity, while RAM- cells controlled by ILF2 and ISL1 promoted metastasis and immune evasion, with RAM- malignant cells enriched in patients over 65 years and associated with immunotherapy resistance. Machine learning analysis of RAM-associated genes identified five core signatures (GPR143, ST3GAL4, RAB38, GMPR, FDFT1) demonstrating superior immunotherapy response prediction (AUC = 0.78-1.00). Drug sensitivity profiling revealed ST3GAL4 exhibited strong correlations with AZ628 (pan-RAF inhibitor) and RDEA119 (MEK inhibitor), which was further validated by molecular docking showing excellent binding affinities (binding energies: -8.7 and − 7.2 kcal/mol). This study provides structural evidence for targeted therapeutic strategies in ST3GAL4-overexpressing melanoma and establishes foundations for age-stratified immunotherapy.

**Supplementary Information:**

The online version contains supplementary material available at 10.1186/s13062-026-00860-x.

## Introduction

Melanoma is strongly associated with biological aging, with a median age at diagnosis of approximately 65 years and incidence rising steeply from the sixth to eighth decades of life [1, 2]. Aging not only increases melanoma risk but also shapes its clinical and biological phenotypes: older patients tend to present with thicker, more ulcerated, less immune-infiltrated, more mitotically active, and more advanced tumors, with age-related differences further extending to metastatic patterns, prognosis, and driver alterations such as BRAFV600E enrichment in younger patients and more frequent NRAS mutations in older cohorts [3–8]. Such age-skewed molecular features may have therapeutic implications, as illustrated by the AVAST-M adjuvant trial, in which bevacizumab improved disease-free intervals in patients younger than 45 years but showed no comparable benefit in older patients [9]. Together, these observations suggest that age shapes melanoma across genetic, stromal, immune, metastatic, and therapeutic dimensions, reflecting the multi-layered nature of biological aging [10]. Mechanistically, aging-related features in melanoma have been linked to CDKN2A alterations, TERT promoter mutations, UV-induced TGFβ/SMAD signaling changes, and global DNA hypomethylation [11–14]. Given that aging is driven by interconnected molecular hallmarks and can be quantified using DNA methylation clocks [15, 16]. Thus, biological aging in melanoma may be better understood as a complex, context-dependent, and cell-type-specific process. Integrative multi-omics analyses are therefore essential for linking aging-associated molecular programs across genomic, epigenomic, transcriptomic, proteomic, and spatial microenvironmental dimensions, enabling a more comprehensive understanding of how aging shapes melanoma biology and therapeutic vulnerability.

The tumor microenvironment (TME) introduces a second layer of age-dependent complexity, as aged stromal cells are increasingly recognized as active drivers of melanoma phenotypes. For example, aged dermal fibroblasts enhance melanoma invasion, angiogenesis, and metastasis by increasing sFRP2 and reducing HAPLN1-dependent ECM organization, while also disrupting immune cell motility and infiltration [17–19]. The immune compartment undergoes parallel remodeling through immunosenescence (thymic involution, TCR contraction, NK/DC dysfunction) [20–22] and inflammaging (IL-6/TNF-α elevation, MDSC expansion) [23], compounded by intratumoral accumulation of senescent MDSCs, macrophages, and endothelial cells [24] - motivating emerging senolytic and senomorphic therapeutic paradigms [25]. Yet despite this mechanistic richness, existing single-cell analyses of melanoma [26–28] have seldom anchored cell-state classifications to quantitatively defined, multi-omics-derived aging signatures, leaving unresolved which TME subpopulations most strongly reflect biological aging and through what communication circuits they reshape tumor behavior.

Immune checkpoint inhibitors (ICIs) have transformed melanoma care - the CheckMate 067 trial established 5-year overall survival of 52% for nivolumab plus ipilimumab versus 44% and 26% for monotherapies [29] - yet durable responses occur in only 40–60% of patients, and predictive biomarkers remain inadequate, with TMB, PD-L1, and composite signatures such as IMPRES and TIDE capturing only a fraction of response heterogeneity and showing limited cross-cohort generalizability [30–32]. Age has been repeatedly nominated as a response modifier, yet the evidence is complex and only partially concordant. Kugel et al. reported that older patients respond better to anti-PD-1, with complete response rates of ~ 47.9% in octogenarians/nonagenarians versus ~ 20% in patients aged 65-79 [33]. Complementing this, prospective profiling of 104 ICI-treated patients revealed that aged and younger patients achieve comparable outcomes through divergent immunological pathways - aged patients exhibit contracted naïve T-cell pools, diminished cytokine responses, and greater reliance on effector T-cell expansion, consistent with immunosenescence-driven remodeling of antitumor immunity [34, 35]. Together, these findings expose the inadequacy of chronological age as a biomarker and motivate integrative computational frameworks capable of capturing the intricate relationships between aging signatures, cellular states, and therapeutic responses-a foundation for genuinely age-informed precision oncology in melanoma.

We applied MOFA [36] to integrate multi-omics melanoma data, including mutation, gene expression, DNA methylation, and copy-number variation, and examined the resulting latent factors for associations with age, survival, and clinical stage. This analysis identified an age-associated axis, termed the RevitalAge Marker (RAM), which was further characterized across cell types, regulatory networks, and intercellular communication using integrated single-cell RNA-seq data. We next trained and externally validated a machine-learning model based on RAM-derived markers for predicting ICI response, and combined drug-sensitivity analysis with molecular docking to identify therapeutic candidates centered on ST3GAL4.Together, these findings identify RAM as an integrative aging-associated framework linked to melanoma cell states, microenvironmental remodeling, and ICI response, underscoring the clinical relevance of biological aging in melanoma stratification.

## Method

### Study design, data resources, and collection of bulk multi-omics and single-cell data

To explore whether melanoma exhibits a coordinated aging-associated program that may relate to tumor-intrinsic alterations, microenvironmental remodeling, and immunotherapy response, we designed a stepwise analytical workflow spanning bulk multi-omics discovery, single-cell resolution mapping, and clinical validation (Figure S1). We first assembled matched multi-omics data from TCGA-SKCM to identify age-associated molecular features across somatic mutation, copy-number variation (CNV), DNA methylation, and transcriptomic layers. We designated the factor6 with the strongest associations with age and clinical outcomes in this analysis as the RevitalAge Marker (RAM). Next, we projected RAM onto integrated single-cell RNA-seq datasets to identify the tumor, stromal, and immune cell states associated with to this aging axis and characterize the associated regulatory and intercellular communication programs. Finally, we evaluated the potential clinical relevance of RAM-associated cell populations using immunotherapy-treated cohorts, bulk deconvolution, response analysis, survival analysis, and downstream therapeutic prioritization.

Bulk multi-omics discovery data were obtained from The Cancer Genome Atlas skin cutaneous melanoma cohort (TCGA-SKCM; https://portal.gdc.cancer.gov/). We retained 465 melanoma samples with complete somatic mutation, CNV, DNA methylation, and mRNA expression profiles. Clinical annotations, including diagnosis age, survival outcome, and tumor stage, were collected for downstream analyses. Somatic mutation data were extracted from TCGA MAF files. We retained non-silent variants, including missense, nonsense, splice-site, frameshift insertion/deletion, in-frame insertion/deletion, translation start site, and nonstop mutations. Gene-level CNV data were obtained from level-3 Affymetrix SNP 6.0 array data and processed using GISTIC2 [37] to estimate focal and broad copy-number alterations. Methylation data were retrieved from the UCSC Xena platform and were based on the Illumina Infinium HumanMethylation450 array. RNA-seq raw count data from TCGA-SKCM were normalized for transcriptomic analysis using the edgeR package [38].

To evaluate the clinical relevance of the identified aging-associated axis in immunotherapy-treated melanoma, we additionally collected the metastatic melanoma transcriptomic cohort PRJEB23709 [39], which included patients treated with anti-PD-1 monotherapy or combined anti-PD-1 and anti-CTLA-4 therapy. For single-cell analysis, we integrated three untreated melanoma datasets (GSE174401, GSE186344, and GSE215121) from GEO and one immunotherapy-treated melanoma single-cell dataset (GSE115978) from TISCH2 [40] database.

### Identification of age-related multi-omics features and MOFA analysis

To reduce dimensionality and enrich for signals relevant to aging, we first identified age-associated features within each omics layer. For mutation data, we focused on non-silent SNVs with mutation frequencies greater than 5% and tested associations with age using logistic regression. For CNV data, copy-number status was encoded as amplification, deletion, or neutral state, and associations with age were evaluated using linear regression. For methylation data, age-associated CpG-linked genes were identified using Spearman correlation analysis. For transcriptomic data, age-related differential expression was assessed using age as a continuous variable.

The age-associated features obtained from these layer-specific analyses were then used as input to MOFA. MOFA was applied to integrate mutation, CNV, methylation, and expression data into a unified latent representation and to identify factors capturing shared variation across omics layers. This two-stage strategy helped us separate layer-specific age signals from cross-omics patterns associated with aging. MOFA analyses were implemented using the MOFA2 package.

### Association of MOFA factors with clinical features and definition of RAM

We next evaluated whether the MOFA-derived latent factors were associated with age, prognosis, and tumor stage. Spearman correlation analysis was used to assess the relationship between factor scores and patient age. An exploratory threshold of ∣Cor∣>0.1 with *P* < 0.05 was used for initial screening of age-associated factors. Among these factors, Factor6 was selected for downstream analyses because it showed the strongest and most consistent clinical relevance. To test whether the age association persisted after adjusting for potential confounders, partial correlation analysis was performed controlling for gender, tumor stage, melanoma Clark level, and non-silent mutations per megabase.

To assess clinical relevance, we fitted univariate Cox proportional hazards models using factor scores as predictors and overall survival as the outcome. Factors significantly associated with survival were retained for further interpretation. We then compared factor scores across tumor stages using the Kruskal-Wallis test to explore whether the same factor also reflected disease progression. The factor6 showing the most robust associations with age, survival, and tumor stage was designated as the RevitalAge Marker (RAM).

### Single-cell preprocessing and cell-state annotation

Single-cell RNA-seq datasets were processed using Seurat v4. Low-quality cells with fewer than 300 detected genes or with mitochondrial transcripts accounting for at least 10% of total gene expression were excluded. The remaining cells were normalized using SCTransform, and the top 3,000 highly variable genes were selected for downstream analysis. To integrate the three untreated melanoma datasets, we used Harmony to correct batch effects across samples. The integrated expression matrix was subjected to principal component analysis, and the leading principal components were used for UMAP visualization and clustering. Clustering resolution was set to 0.4. Cell identity annotation was performed using SingleR [41] as an initial guide, followed by marker-based refinement using FindAllMarkers in Seurat and literature-supported canonical markers.

### Mapping RAM-associated cells in single-cell data

To localize the bulk-derived RAM signal to specific cellular populations, we applied the Scissors algorithm to the integrated single-cell and bulk transcriptomic datasets [42]. Scissors integrates single-cell expression profiles, bulk expression profiles, and matched phenotype annotations. The algorithm first computes a correlation matrix between each single cell and each bulk sample to estimate transcriptomic similarity. It then applies phenotype-specific regression modeling to this correlation matrix, supporting continuous, binary, and survival phenotypes. Based on the fitted model, individual cells are classified as positively associated cells, negatively associated cells, or background cells with respect to the phenotype of interest.We implemented RAM-related cell identification using the “Scissor” R package, with input data including integrated single-cell expression profile data, TCGA expression profile data, and senescence phenotype scores of TCGA samples, with parameters alpha = 0.05, family = “gaussian”.

We identified cell-specific marker genes in different senescence phenotype cells through differential expression analysis using the non-parametric Wilcoxon rank-sum test with the FindMarkers function, and performed enrichment analysis on these marker genes using the “ClusterProfiler” package [43]. Additionally, we downloaded Hallmark gene set feature files from MSigDB [44] (https://www.gsea-msigdb.org/gsea/msigdb/).We further used the “AUCell” package to score the activity of Hallmark pathways and REACTOME metabolic pathways in malignant cells, and used the “limma" [45] package to compare pathway activation differences between RAM+ cells and RAM- cells.

### Analysis of cell subpopulation distribution preferences

To comprehensively understand the distribution preferences of cell types within the RAM groups, we calculated the ratio (Ro/e) between observed and expected cell counts for each cluster [46], where ‘o’ represents the observed cell count and ‘e’ represents the expected cell count. The expected cell counts were determined through chi-square testing, which was employed to verify significant associations between two categorical variables. In this context, the chi-square test was utilized to confirm the expected distribution of specific cell types across RAM groups, implemented using the chisq.test() function in R. When the Ro/e value exceeds 1, it indicates that the observed cell count surpasses the expected level. This suggests an enrichment of that particular cell type within a specific group, meaning that relative to other groups, this cell type appears in higher numbers within that group. The calculation formula is as follows:

$$\:Ro/e=\frac{observed}{excepted}$$

To account for patient-level structure and reduce potential pseudoreplication, we further performed patient-level compositional analysis. For each patient, the fraction of each annotated cell type was calculated separately among RAM + and RAM- cells. RAM + and RAM- cell-type fractions were then compared across patients using paired Wilcoxon signed-rank tests, followed by Benjamini-Hochberg correction for multiple testing. Cell types with FDR < 0.05 were considered statistically significant.To evaluate whether RAM-associated cell-type composition patterns exceeded random expectation, we performed a label-permutation analysis. RAM labels were randomly shuffled 1,000 times across cells while preserving the total numbers of RAM + and RAM- cells and keeping cell-type annotations unchanged. For each permutation, the observed-to-expected cell number ratio Ro/e was recalculated for each annotated cell type within each RAM group. Empirical null distributions of log2(Ro/e) were generated from the 1,000 permutations. Empirical P values were calculated as the proportion of permutations with absolute log2(Ro/e) values greater than or equal to the observed absolute log2(Ro/e), followed by Benjamini-Hochberg FDR correction. Empirical P values of 0 were reported as *P* < 0.001, corresponding to the permutation resolution.

### Cell-cell communication and transcriptional regulation analysis

We employed the R package “CellChat " to analyze cell-cell interactions. CellChat incorporates a comprehensive database of signaling molecule interactions, taking into account receptor-ligand interactions with known structural compositions [47]. We further utilized Single-Cell Regulatory Network Inference and Clustering (SCENIC) to determine the regulatory networks of malignant cells [48]. The SCENIC workflow reveals co-expression patterns between transcription factors and potential target genes, utilizing transcription factor motifs to identify target genes. To quantify the specific correspondence between regulons and each cell type, we conducted SCENIC analysis using pySCENIC in Python and calculated regulon specificity scores using the regulon_specificity_scores function. This function employs an entropy-based strategy to analyze regulon expression patterns across different cell types, generating Regulon Specificity Scores (RSS) [49], thereby quantifying the specific activity levels of regulons across different cell types.

### Deconvolution of bulk immunotherapy cohorts and clinical validation

To test whether RAM-associated cell states were clinically relevant in immunotherapy-treated melanoma, we used CIBERSORTx to deconvolve bulk transcriptomes using single-cell-derived reference signatures. Single-cell expression matrices from melanoma were used to construct a reference signature matrix, and this matrix was applied to the PRJEB23709 metastatic melanoma cohort to estimate the relative abundance of 30 tumor-infiltrating cell populations in each sample.

We then compared immune cell proportions between responders and non-responders using the Wilcoxon rank-sum test. To further evaluate prognostic relevance, samples were stratified into high- and low-infiltration groups based on the median abundance of each cell population, and survival curves were generated using the Kaplan-Meier method. Group differences were assessed with the log-rank test.

### Drug prioritization and molecular docking

Protein information was obtained from the UniProt website (https://www.uniprot.org/uniprotkb), and protein structures were retrieved from the RCSB PDB database (https://www.rcsb.org/). The obtained structures were imported into Discovery Studio 2019 for protein structure optimization. This process mainly included removing water molecules, adding hydrogen atoms, completing charges, completing amino acids, and completing side chains. Finally, the optimized protein structure was obtained and exported as a PDB file.

Small molecule structures were obtained from the PubChem website and subjected to energy minimization using Discovery Studio 2019, then saved as PDB files. AutoDock 1.5.6 was used to prepare the protein and small molecule PDBQT files, and AutoDock Vina 1.2.6 was used for docking. Finally, visualization analysis was performed in PyMOL 3.1 and Discovery Studio 2019.

## Result

### Result1: MOFA-derived factor6 as an age-dependent survival protective factor in melanoma

**Fig. 1:**
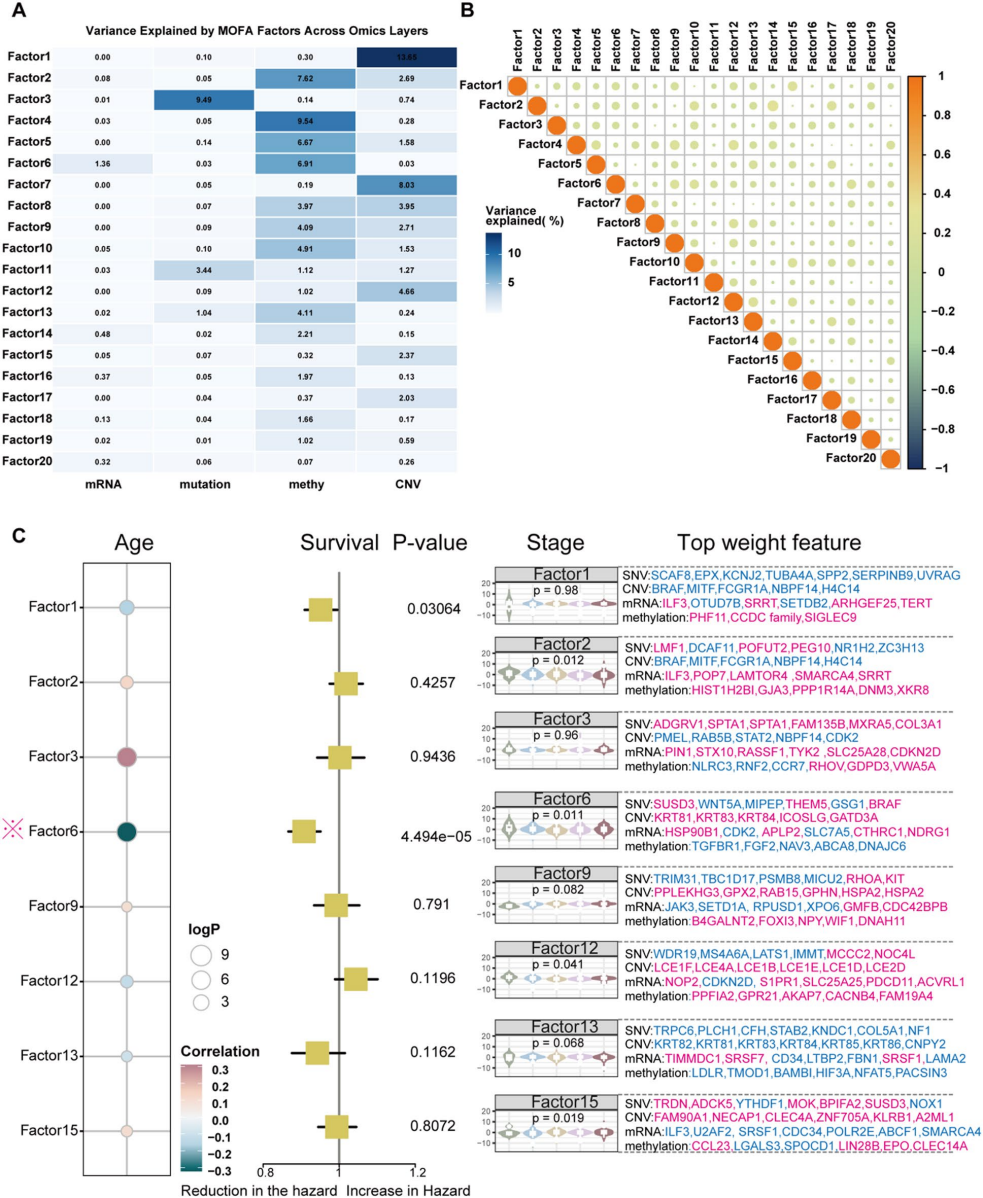
MOFA factor analysis reveals multi-omics variance patterns and age-associated clinical correlations: **A-B**. The factors generated by the MOFA model enable variance decomposition and correlation analysis in multi-omics data. **C**. Correlation analysis of factors with age, survival, and staging, as well as multi-omics characteristics of factors. Blue indicates downregulated features and red, upregulated

Based on the multi-omics age-related analysis, we constructed age-related profiles of mutation, mRNA expression, DNA methylation, and copy number variation. We then trained a MOFA model using these four omics layers, which identified 20 latent factors representing major sources of molecular variation across the integrated dataset. Each factor was associated with at least one omics feature. Among these factors, Factor 1 accounted for 13.65% of the variance in copy number variation; Factor 2 accounted for 7.62% of the variance in DNA methylation and 2.69% of the variance in copy number variation; and Factor 3 accounted for 9.49% of the variance in mutation features (Fig. 1A). The MOFA factor scores represent sample-level latent scores inferred from the integrated multi-omics model and reflect the extent to which each latent factor contributes to molecular variation across samples. Next, we calculated the Pearson correlations between different factor scores and found that the correlation coefficients (Cor) were all less than 0.2, indicating their relative independence in explaining data variation and features (Fig. 1B). We further analyzed the correlations between these 20 latent factors and age as well as other clinical features. We found that Factor1, Factor2, Factor3, Factor6 and Factor12 were significantly correlated with age (Fig. 1C, Table S1). Next, we explored the correlations between these age-associated latent factors and other clinical features. Using univariate Cox regression, we evaluated the association between factor sample scores and survival status and time. We found that Factor1 (HR = 0.91, P-value = 0.031) and Factor6 (HR = 0.95, P-value = 4.494e-05) were survival-related latent factors and acted as protective factors (Fig. 1C). Additionally, we found that Factor2, Factor6, Factor12, and Factor15were significantly associated with cancer stage (Fig. 1C).

We focused on Factor 6, which showed a significant negative correlation with age and was associated with favorable melanoma prognosis in survival analysis. These findings suggest that Factor 6 may reflect a molecular signature of a younger state and better clinical outcome. To further characterize its omics composition, we examined the variance explained by Factor 6 across molecular layers. Factor 6 captured a major source of cross-omics variation in the integrated dataset and was primarily driven by DNA methylation, accounting for 6.9144% of the variance in methylation features. It also explained 1.36086% of the variance in mRNA expression, whereas mutation and copy number variation contributed minimally, accounting for 0.0305% and 0.0284% of the variance, respectively. These results indicate that Factor 6 was primarily methylation-enriched, with additional transcriptomic support, rather than being uniformly contributed by all omics layers. This pattern may reflect that aging-associated molecular variation is more readily captured by epigenetic and transcriptional remodeling than by structural genomic alterations such as mutations or CNVs, because epigenetic and transcriptional changes are more dynamic and tend to accumulate more rapidly than fixed genetic alterations [50]. To further evaluate whether this age association was independent of potential clinical and molecular confounders, we performed covariate-adjusted analyses. In multivariable linear regression adjusting for sex, tumor stage, melanoma Clark level, and non-silent mutations per megabase, chronological age remained significantly associated with Factor 6, with an estimated coefficient of − 0.0507 per year (FDR < 0.001; Figure S2A). Consistently, partial correlation analysis controlling for the same covariates confirmed a significant negative association between Factor 6 and age (partial *r* = − 0.257, *p* < 0.001). Multivariable Cox regression further showed that higher Factor 6 values were independently associated with better overall survival, suggesting that Factor 6 is an independent prognostic factor after adjustment for the same covariates (Figure S2B). Based on these findings, we termed Factor 6 the RevitalAge Marker (RAM), defined as an epigenetically enriched, age-associated latent factor identified within the integrated MOFA framework.

To further characterize the molecular features underlying RAM, we examined high-weight multi-omics features contributing to this factor (Fig. 1C). Feature weights indicate the contribution of each molecular feature to the latent factor: positive weights represent features that increase with higher RAM scores, whereas negative weights represent features that decrease with higher RAM scores. Methylation features were mainly linked to genes involved in growth factor signaling, tissue remodeling, and stress responses, including TGFBR1, FGF2, and DNAJC6. Transcriptomic features included genes associated with proteostasis, cell-cycle regulation, and stress adaptation, such as HSP90B1, CDK2, and NDRG1. Mutation- and CNV-associated features further suggested potential involvement in mitochondrial metabolism, Wnt-related signaling, and immune regulation, including MIPEP, THEM5, WNT5A, and ICOSLG. Collectively, RAM appears to represent a health-maintenance module characterized primarily by proteostasis, cell-cycle regulation, mitochondrial homeostasis, and tissue repair, with a possible contribution from immune-related regulation through ICOSLG. Its depletion may therefore reflect a core aging process marked by a shift from active physiological repair and regeneration toward functional quiescence and epigenetic repression.

## Result2: Single-cell analysis reveals RAM factor-associated cellular heterogeneity and functional specialization in melanoma microenvironment

**Fig. 2:**
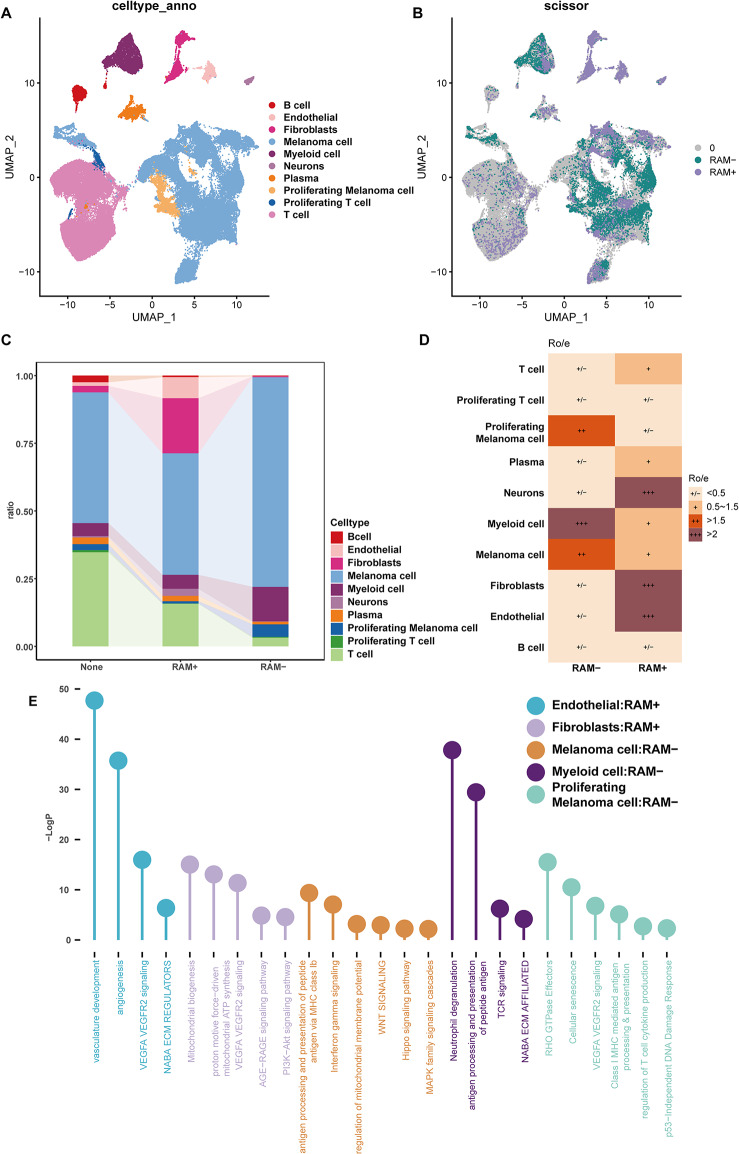
Cell type-specific distribution and functional enrichment analysis of aging-associated RAM phenotypes. **A-B**: Single-cell annotation and The UMAP visualization of the Scissors selected cells. **C**. The distribution of cell type proportions among different groups. **D**. Heatmap showing tissue prevalence for each cell type by Ro/e score. **E**. GO term enriched by marker genes in different cells

We further analyzed the tumor microenvironment associated with the aging-related RAM factor at single-cell resolution. We integrated three melanoma single-cell datasets, GSE174401, GSE186344, and GSE215121, and identified 23 clusters after cell clustering. These clusters were annotated into 10 cell subpopulations, including B cells, endothelial cells, fibroblasts, myeloid cells, T cells, proliferating T cells, plasma cells, neuronal cells, melanoma cells, and proliferating melanoma cells (Fig. 2A). Next, based on the SCISSORS algorithm, we identified cells associated with the aging phenotype: 4,796 RAM+ cells, potentially linked to a reduced risk of aging-related tumor deterioration, and 7,229 RAM- cells, which may be associated with an increased risk of aging-related tumor deterioration. The remaining cells were classified as background cells (Fig. 2B). The remaining cells were designated as background cells. We observed differences in overall cell-type composition among background, RAM-, and RAM+ cells (Fig. 2C). For example, RAM- cells contained a higher proportion of malignant cells, whereas RAM+ cells showed a higher proportion of fibroblasts.

To assess RAM-associated cell-type composition, we calculated the observed-to-expected ratio (Ro/e) for each cluster as a descriptive measure. Ro/e suggested enrichment of melanoma-related and myeloid cells in RAM- cells, and endothelial cells and fibroblasts in RAM+ cells (Fig. 2D). We then performed 1,000 RAM-label permutations to compare the observed Ro/e values with the random-label null distribution. This analysis indicated that endothelial cells, fibroblasts, neurons, T cells, plasma cells, and B cells were preferentially enriched in RAM+ cells, whereas proliferating melanoma cells, myeloid cells, melanoma cells, and proliferating T cells were preferentially enriched in RAM- cells (Table S2). These findings suggest that RAM-associated cell-type composition is unlikely to arise from random cell grouping and may reflect underlying biological structure.Given the potential influence of unequal patient sampling and pseudoreplication in pooled single-cell analyses, we further performed a patient-level cell-type compositional analysis. After FDR correction, T cells, fibroblasts, and endothelial cells were significantly more abundant in the RAM+ group, whereas myeloid cells and melanoma cells were significantly more abundant in the RAM- group (Figure S3). These patient-level results support the robustness of the major RAM-associated compositional patterns while reducing the potential impact of pooled-cell sampling bias.

Since RAM decreased with chronological age and was independently associated with favorable survival, we next investigated its cellular basis at the single-cell level. We used FindMarkers to compare differentially expressed genes between RAM + and RAM- cells across major cell types, identifying genes upregulated in RAM+ endothelial cells and fibroblasts, as well as genes upregulated in RAM- melanoma cells, myeloid cells, and proliferative melanoma cells (Fig. 2E). RAM-defined cell states showed distinct functional programs across the melanoma microenvironment. RAM+ states were mainly associated with vascular-stromal organization and mitochondrial metabolic activity, processes broadly involved in tissue remodeling during aging and cancer progression [51]. In RAM+ endothelial cells, upregulated genes were enriched for vasculature development, angiogenesis, VEGFA-VEGFR2 signaling, and NABA ECM regulators, suggesting a program related to vascular organization and extracellular matrix remodeling, consistent with the established role of VEGF-VEGFR signaling in angiogenesis and vascular remodeling [52]. RAM+ fibroblasts were enriched for mitochondrial biogenesis, proton motive force-driven mitochondrial ATP synthesis, and AGE-RAGE signaling, indicating a stromal state linked to mitochondrial energy metabolism and stress-associated signaling [53]. Together, these RAM+ programs point to tissue organization and metabolic homeostasis, two processes frequently remodeled during aging [10]. In contrast, RAM- states were characterized by inflammatory, oncogenic, immune-related, and proliferative programs. RAM- melanoma cells were enriched for interferon-gamma signaling, regulation of mitochondrial membrane potential, WNT, Hippo, and MAPK signaling, suggesting immune-related, stress-response, and tumor-associated activity. RAM- myeloid cells showed enrichment of antigen processing and presentation and TCR-related signaling, indicating altered immune-interaction programs. RAM- proliferative melanoma cells were enriched for regulation of T-cell cytokine production and RHO GTPase effector pathways, suggesting a proliferative state linked to immune modulation and cytoskeletal signaling.

**Fig. 3:**
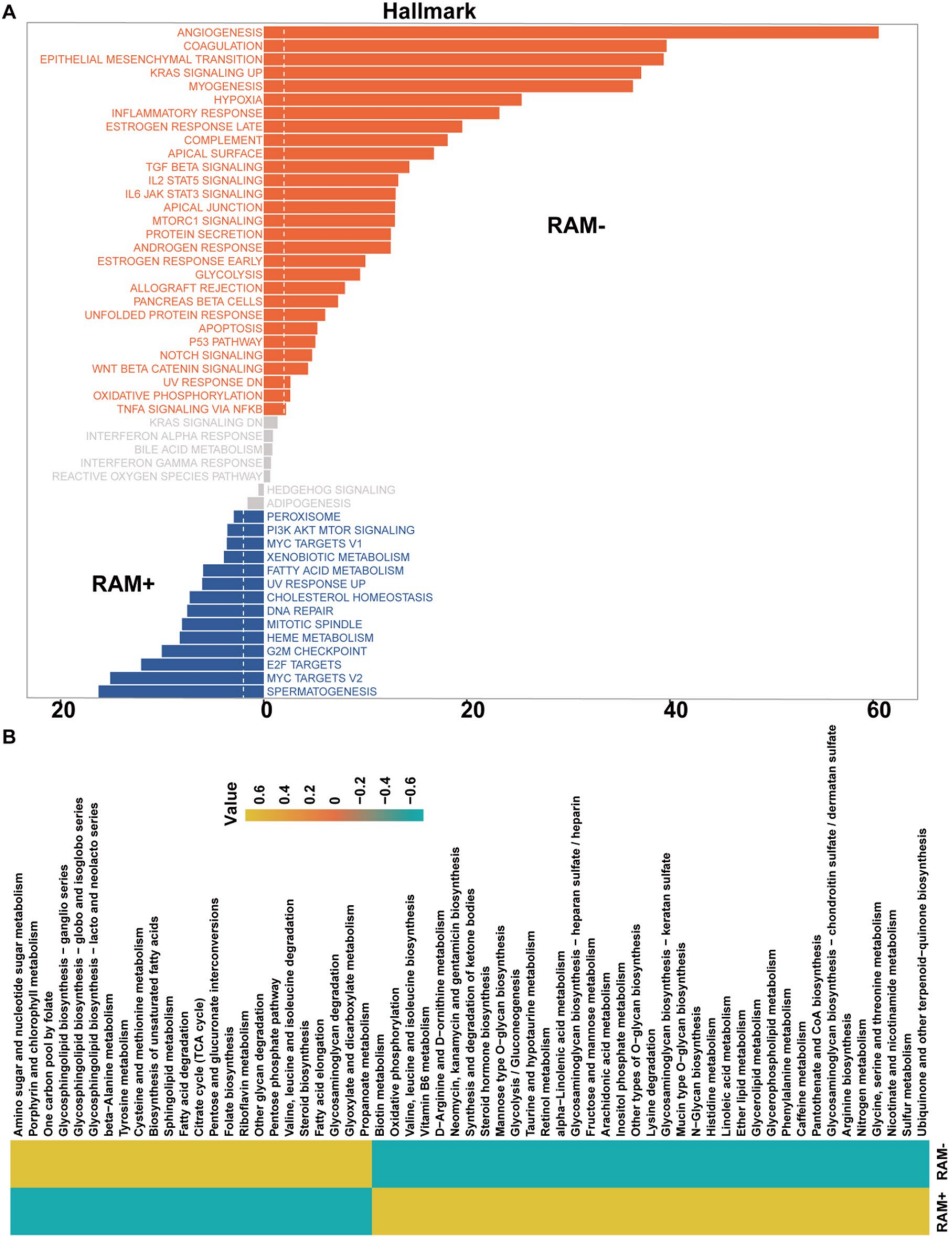
Differential Pathway Activity Analysis in RAM-Associated Malignant Melanoma Cell Populations. **A** The difference in pathway activity scores of RAM+ malignant melanoma cells RAM- malignant melanoma cells evaluated by Aucell for each cell. **B** Heatmap showing significant changes in metabolic pathways of RAM+ malignant melanoma cells and RAM- malignant melanoma cells

To further characterize RAM phenotype-associated malignant cells, we compared Hallmark pathway activities between RAM + and RAM- malignant cells (Fig. 3A). RAM- malignant cells exhibited higher activity of multiple malignancy-associated Hallmark pathways, including angiogenesis, epithelial-mesenchymal transition, and hypoxia. Although angiogenesis-related enrichment was observed in both RAM+ endothelial cells and RAM- malignant cells, these signals may reflect different cellular contexts. In RAM+ endothelial cells, angiogenesis-related pathways may represent stromal vascular organization and endothelial remodeling, whereas in RAM- malignant cells, angiogenesis together with hypoxia may reflect a tumor-cell-intrinsic stress-adaptive program. The concurrent enrichment of epithelial-mesenchymal transition and hypoxia in RAM- malignant cells further supports their association with invasive and stress-adaptive malignant phenotypes.We next evaluated REACTOME metabolic pathway activity in RAM + and RAM- malignant cells (Fig. 3B). RAM+ malignant cells showed higher activity of nicotinate and nicotinamide metabolism, which is closely related to NAD metabolism, as well as ubiquinone and other terpenoid-quinone biosynthesis. These metabolic pathways are linked to redox balance, mitochondrial electron transport, and cellular bioenergetic homeostasis. Therefore, compared with RAM- malignant cells, RAM+ malignant cells may exhibit a metabolic profile characterized by relatively greater mitochondrial and redox homeostasis.

Collectively, these single-cell analyses suggest that RAM-defined cellular states provide a functional link between age-associated multi-omics variation and cell-type-specific remodeling of the melanoma microenvironment. Overall, RAM+ states were associated with vascular-stromal organization, extracellular matrix remodeling, and mitochondrial metabolic homeostasis, whereas RAM- states were linked to inflammatory activation, WNT/MAPK/Hippo signaling, hypoxia, epithelial-mesenchymal transition, immune interaction, and proliferative tumor-cell programs. Given that RAM decreases with age, these pathway-level differences may reflect age-associated remodeling of the melanoma microenvironment.

## Result3: RAM-associated remodeling of intercellular signaling and regulatory networks in the melanoma microenvironment

**Fig. 4:**
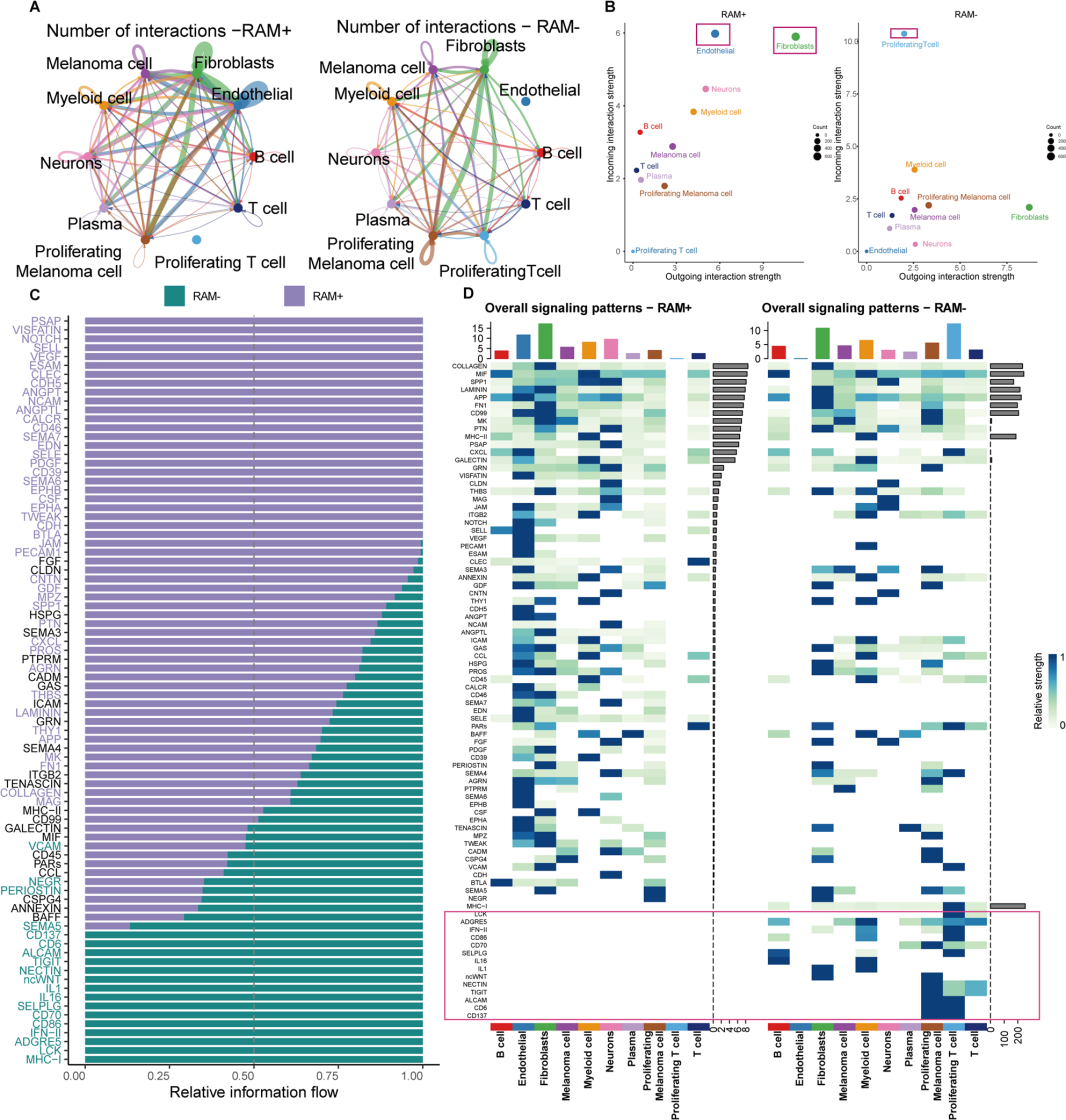
Cell communication networks and signaling pathway analysis in RAM + and RAM- Groups: **A**. Cell communication within RAM + and RAM- groups. **B**. Incoming and outgoing strength of each cell in RAM + and RAM-. **C**. The relative information flow in intracellular signaling pathways between the RAM + and RAM- groups. **D**. Signal Pathway Comparisons between Cell Types in RAM + and RAM- Groups

We compared cell-cell communication between RAM + and RAM- groups using CellChat. Edge thickness in Fig. 4A indicates interaction strength. Heatmaps showed differential incoming and outgoing signaling across cell types, with red indicating increased and blue indicating decreased signaling in RAM+ relative to RAM-. Endothelial cells exhibited stronger interactions in RAM+, whereas proliferative melanoma cells showed stronger interactions in RAM- (Figure S4). Comparison of incoming and outgoing communication strengths revealed that proliferative melanoma cells exhibited lower outgoing signaling in both groups but higher incoming signaling in RAM- cells. Fibroblasts showed higher outgoing signaling in both groups and higher incoming signaling in RAM+ cells. Endothelial cells displayed low interaction intensity in RAM- cells but higher intensity in RAM+ cells. Together, these results indicate that RAM status is associated with distinct intercellular communication patterns within the tumor microenvironment, with stromal and malignant compartments displaying different signaling roles across the two groups.

Analysis of total communication probability across signaling pathways revealed distinct activity patterns between RAM- and RAM+ conditions (Fig. 4C). Several pathways showed preferential activity in RAM+, whereas others were more active in RAM- cells, and a subset remained comparably active in both groups. Notably, MIF, LAMININ, and COLLAGEN pathways showed consistently high signaling activity regardless of RAM status. The MIF pathway has been implicated in immune regulation, inflammatory responses, and oncogenic processes [54]. In contrast, LAMININ and COLLAGEN pathways may reflect conserved extracellular matrix-related communication and structural interactions across RAM states. The preserved activity of these pathways suggests that ECM-associated signaling and MIF-related immune/inflammatory signaling represent prominent inferred communication axes in both groups, despite broader differences in pathway usage.

Further analysis of outgoing and incoming signals across cell types revealed distinct pathway activation patterns between RAM- and RAM+ groups (Fig. 4D). Pathways preferentially active in the RAM- group were mainly observed in proliferative melanoma cells and proliferative T cells. This pattern suggests that signaling activity in the RAM- group is more strongly distributed within proliferative tumor and immune compartments. In particular, TIGIT-related signaling was selectively enriched in these cell subsets, indicating a potential association with inhibitory immune signaling in the RAM- microenvironment. By contrast, RAM+ samples showed relatively stronger signaling activity in stromal-associated compartments, including endothelial cells and fibroblasts. These differences suggest that RAM status is linked to a redistribution of signaling activity across cell types, with with distinct inferred communication patterns observed in RAM + and RAM- tumors. Overall, the differential pathway activity between RAM- and RAM+ groups highlights the heterogeneity of inferred signaling states and suggests that RAM status is associated with distinct cell-cell signaling states in melanoma.

In conclusion, our intercellular communication analysis suggests RAM-associated differences in inferred cell-cell communication within the tumor microenvironment. Compared with RAM+, RAM- samples showed increased signaling activity in malignant and proliferative compartments, whereas RAM+ samples exhibited relatively stronger stromal-associated interactions. The selective activity of TIGIT-related signaling in RAM- proliferative melanoma and proliferative T-cell subsets further suggests that inhibitory immune signaling may be a feature of the RAM- microenvironment [55]. Collectively, these results indicate that RAM status is associated with distinct intercellular communication networks and signaling pathway activity in melanoma.

**Fig. 5:**
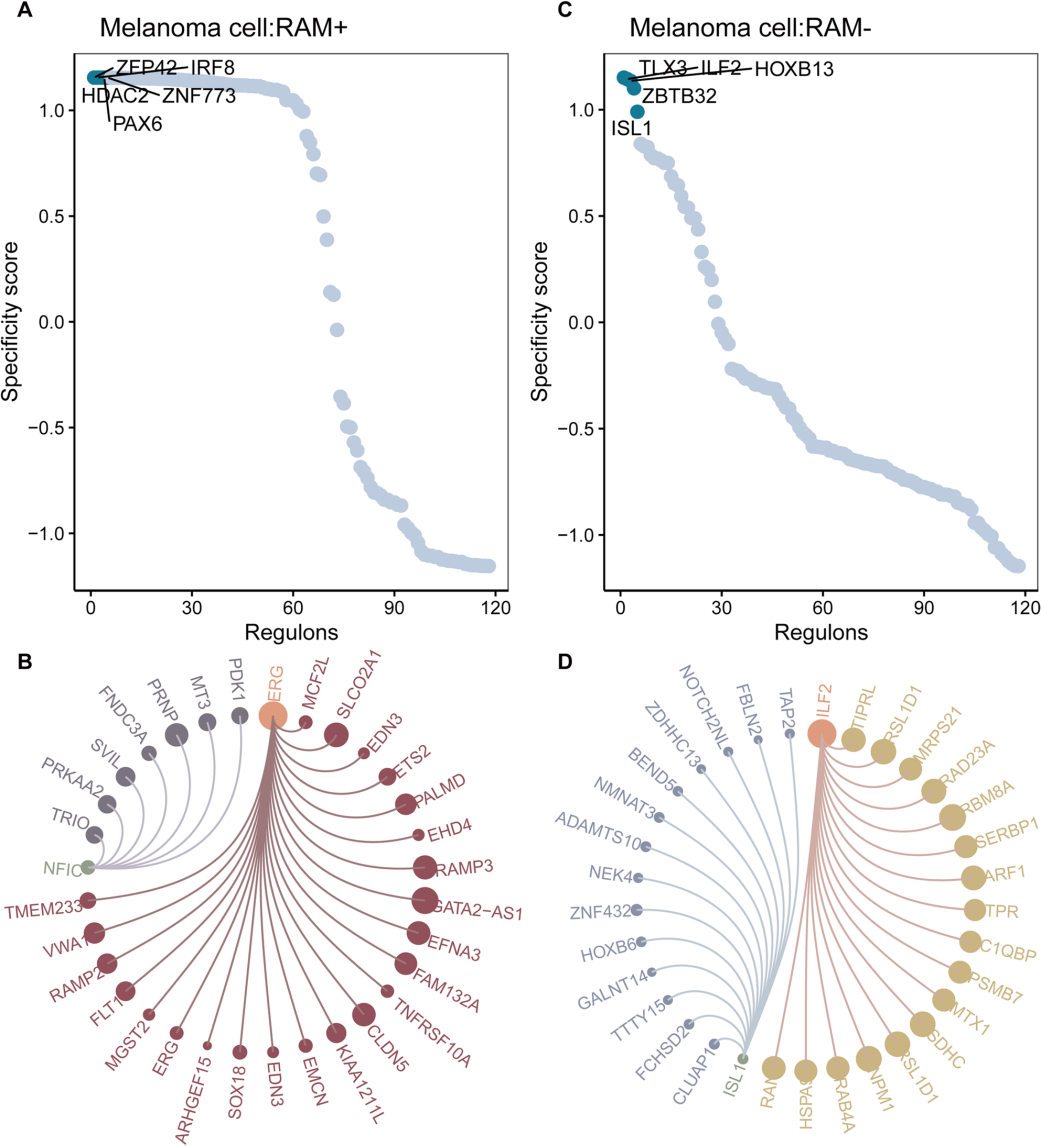
Transcriptional regulatory networks and gene target analysis in RAM+ versus RAM- cells: **A** and **C** Transcription factors identified by SCENIC in the RAM + and RAM-, cells. **B** and **D** The relationship between transcription factors network with target genes

We performed SCENIC analysis on RAM+ cells, RAM- cells, and background cells in melanoma malignant cells, identifying the top five differentially activated transcription factors in each group. In RAM+ cells, the top differentially activated regulons included ZFP42, IRF8, HDAC2, PAX6, and ZNF773. ZFP42 (Fig. 5A), ZFP42, a stem cell-associated transcription factor, has been reported to exert tumor-suppressive roles in some cancer contexts [56]. IRF8 has been implicated in tumor immune regulation and anti-tumor response-associated transcriptional programs [57].Analysis of ZFP42- and IRF8- associated transcriptional networks suggested enrichment of genes related to angiogenesis and metabolic pathways in RAM+ cells, .suggesting a paradoxical role with both tumor suppressive and pro-angiogenic capabilities in melanoma progression (Fig. 5B).In RAM- cells, we identified TLX3, ILF2, HOXB13, and ZBTB32 as differentially activated transcription factors (Fig. 5C). ILF2 promotes metastatic melanoma cell proliferation and DNA damage response [58], while HOXB13 shows elevated expression in metastatic melanoma patients. Aberrant ISL1 expression has been associated with cancer development and has been reported to promote melanoma cell proliferation and invasion through epithelial-mesenchymal transition [59]. We constructed a gene regulatory network using differentially expressed genes and transcription factors ILF2 and ISL1 (Fig. 5D). This network involves molecules related to autophagy (TIPRL), mitochondrial energy metabolism (MRPS21, MTX1, SDHC), and transmembrane transport (ARF1, RBM8A, SERBP1). The distinct regulon activity patterns between RAM + and RAM− groups suggest different transcriptional regulatory states in melanoma malignant cells.

In summary, SCENIC analysis revealed distinct RAM-associated transcriptional regulatory programs in melanoma malignant cells. RAM+ cells showed increased activity of ZFP42- and IRF8-associated regulons, which may be linked to tumor-suppressive and immune-related transcriptional features. Conversely, RAM− cells were characterized by increased activity of ILF2- and ISL1-associated regulatory programs, with potential links to stress adaptation, DNA damage response, and EMT-associated processes. These findings provide potential clues to the regulatory differences underlying RAM-associated malignant cell states in melanoma.

### Result4: RAM phenotype-associated cellular subpopulations predict immunotherapy response and survival outcomes in melanoma

**Fig. 6:**
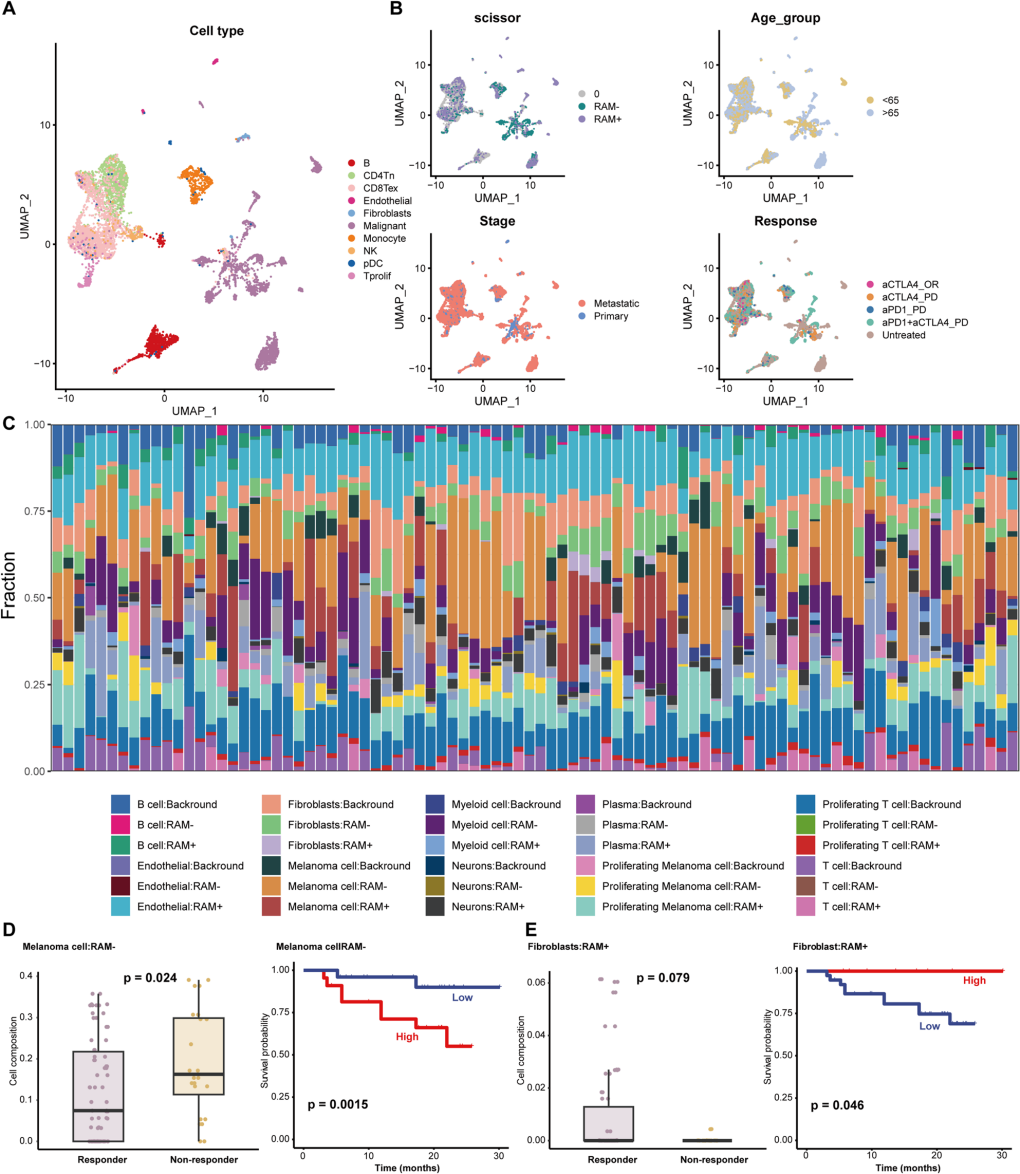
Role and clinical significance of aging phenotype-associated cellular subpopulations in immunotherapy: **A**. The UMAP visualization of GSE115978 scRNA-seq dataset. **B**. Scissors identification results and clinical information of GSE115978. **C**. Immune cell infiltration analysis under RAM grouping in PRJEB23709. **D**-**E**. The role of RAM- malignant cells and RAM+ fibroblasts in immunotherapy

To investigate the association between aging phenotype-related cellular subpopulations and immunotherapy exposure or response, we analyzed the GSE115978 single-cell dataset from TISCH2.0, which contains melanoma samples from 15 patients who did not receive immunotherapy and 16 patients who underwent immunotherapy (Fig. 6A). The dataset also includes cell annotation information (Fig. 6B) and relevant clinical variables, such as age and disease stage. We applied the SCISSOR algorithm to identify cellular subpopulations associated with aging-related phenotypes.RAM+ cells were enriched for immune populations, including CD4Tn cells, CD8Tex cells, and B cells, whereas malignant cells were less prevalent. By contrast, RAM- cells showed higher proportions of malignant cells and monocytes, together with fewer immune cells (Figure S5A-B). These findings suggest that RAM status is associated with distinct immune compositions within the tumor microenvironment.We further assessed the relationship between RAM status and patient age. Given that the average age at diagnosis for melanoma is approximately 65 years, patients were stratified into two age groups using 65 years as the cutoff. Fisher’s exact test showed that RAM- cells were significantly enriched in patients older than 65 years (*P* < 2.2e-16), indicating that RAM- cells were more frequently observed in older patients in this cohort (Figure S5C).

Next, cells were classified into RAM+, RAM-, and background groups. We then compared the inferred number and strength of cell-cell communications between immunotherapy-treated and untreated samples. In post-treatment samples, CellChat analysis indicated enhanced L1CAM-related communication involving RAM- malignant cells (Figure S6). Previous studies have linked L1CAM to immunosuppressive signaling, including CCL22-mediated recruitment of regulatory T cells and TGF-β-associated immunosuppression [60]. Thus, the observed L1CAM-related communication in RAM- malignant cells may indicate a potential association with treatment-adaptive or immunosuppressive communication programs under immunotherapeutic pressure. Further functional validation is needed to determine whether L1CAM directly contributes to immune evasion in this context.

We further analyzed bulk transcriptome data from immunotherapy cohort PRJEB23709 to infer the relative proportions or activity levels of 30 cell types under different RAM groups (Fig. 6C). Based on the cell type preferences in RAM groups, we focused on the roles of RAM+ fibroblasts and RAM- malignant cells in immunotherapy. We found that non-responders had a higher estimated abundance of RAM− malignant cells (Fig. 6D), while responders had a higher proportion of RAM+ fibroblasts (Fig. 6E). This suggests that the proportion of aging-related cell subpopulations may be associated with immunotherapy efficacy. Additionally, a high proportion of RAM- malignant cells and a low proportion of RAM+ fibroblasts were significantly associated with poorer overall survival (Fig. 6D-E). These findings indicate that RAM phenotype-related cell types in the tumor microenvironment are associated with patient survival. The association between aging phenotype RAM-related cell subpopulations, immunotherapy response, and patient prognosis provides a basis for further investigation of RAM-associated mechanisms underlying immunotherapy response.

## Result5: Machine learning-derived RAM cell markers as biomarkers for melanoma immunotherapy response

**Fig. 7:**
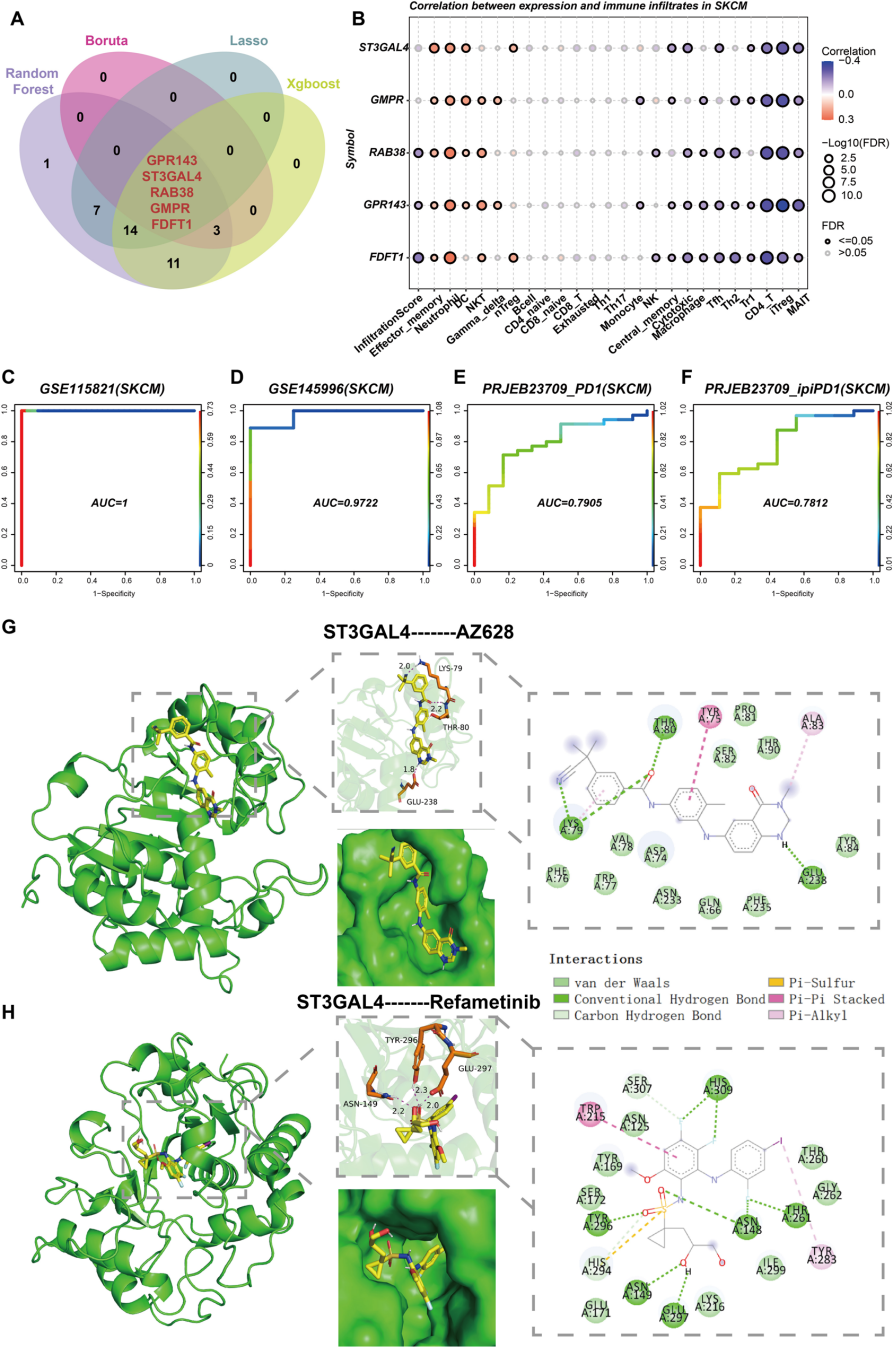
Development and validation of RAM cell marker-based predictive model for melanoma immunotherapy response. (**A**) Feature selection using multiple machine learning algorithms. (**B**) Correlation analysis between RAM cell marker genes and immune cell infiltration in SKCM. (**C**-**F**) ROC curve analysis for immunotherapy response prediction across independent melanoma cohorts. (**G**-**H**) Molecular docking showing the binding modes of AZ628 (**G**) and Refametinib (**H**) with ST3GAL4 protein. Each panel set includes: three-dimensional protein structure (left), detailed view of ligand binding pocket (middle), and two-dimensional interaction diagram (right). Interaction types include van der Waals forces, hydrogen bonds, Pi-Pi stacked, and Pi-alkyl interactions

We developed a predictive model for immunotherapy response in melanoma patients. Through integrating four machine learning algorithms (Boruta, Lasso, Random Forest, and Xgboost) for feature selection from RAM-cell marker genes, we identified 5 core predictive genes: GPR143, ST3GAL4, RAB38, GMPR, and FDFT1 (Fig. 7A). These genes demonstrated consistent importance across multiple algorithms, with 14 features being commonly selected, indicating robust predictive potential for immunotherapy response.

Correlation analysis between the five RAM cell marker genes and immune cell infiltration in Skin Cutaneous Melanoma (SKCM) revealed significant associations with the tumor immune microenvironment (TIME) (Fig. 7B). Notably, ST3GAL4 exhibited robust negative correlations with multiple immune cell types, whereas GPR143, RAB38, GMPR, and FDFT1 showed distinct correlation patterns with specific immune subsets (*p* < 0.05). These findings suggest that RAM markers play pivotal roles in reshaping the immune landscape and influencing immunotherapy responsiveness. To validate the predictive capability of our RAM cell marker-based model for immunotherapy response, we performed ROC curve analysis across four independent melanoma cohorts (Fig. 7C). The model achieved AUC values of 1.000 in the GSE115821 dataset, 0.9722 in the GSE145996 dataset, and 0.7905 and 0.7812 in the PRJEB23709_PD1 and PRJEB23709_ipiPD1 cohorts, respectively. These findings demonstrate that RAM cell marker genes can effectively predict immunotherapy response in melanoma patients. The strong predictive performance across multiple independent cohorts, particularly in PD-1 and combination immunotherapy datasets, suggests that these RAM cell markers serve as reliable biomarkers for identifying patients most likely to benefit from immune checkpoint inhibitor therapy.

We employed machine learning algorithms to screen core genes and integrated them with the GDSC (Genomics of Drug Sensitivity in Cancer) database for drug sensitivity correlation analysis to explore the potential of these core genes as therapeutic targets and predictive biomarkers. We specifically focused on negatively correlated drugs, where high expression of core genes was associated with increased drug sensitivity, indicating better therapeutic efficacy in patients with high expression levels (Figure S7). The RAM signature was associated with predicted sensitivity to Dabrafenib and Trametinib., the current standard-of-care treatment regimen, supporting the possibility that the model captures transcriptional features related to MAPK pathway-associated drug response in melanoma. Building on this robust validation, ST3GAL4 exhibited strong sensitivity correlations with AZ628 (a potent pan-RAF inhibitor) and RDEA119 (Refametinib, a MEK inhibitor), suggesting these drugs as promising novel therapeutic strategies for ST3GAL4-overexpressing tumors. xternal dataset validation showed that ST3GAL4 was significantly upregulated in TCGA-SKCM tumors compared with GTEx normal skin tissuess (Figure S8A), with Wilcoxon *p* < 2.2 × 10^-16. High ST3GAL4 expression was also associated with poorer survival in two untreated melanoma cohorts, GSE65904, with HR = 1.509 and *p* = 0.044 (Figure S8B), and GSE98394, with HR = 6.683 and *p* < 0.0001(Figure S8C), supporting its potential prognostic relevance in melanoma. Mechanistically, ST3GAL4 (β-galactoside α-2,3-sialyltransferase) plays a critical role in melanoma progression and is typically associated with poor prognosis and metastasis; through sialylation modifications of cell surface glycans, ST3GAL4 generates “don’t eat me” signals that promote immune evasion, highlighting its potential as a key therapeutic target [61]. To validate this prediction, we performed molecular docking analysis (Fig. 7G-H): AZ628 demonstrated excellent binding affinity with ST3GAL4 protein (binding energy of -8.7 kcal/mol), primarily achieving specific recognition through conventional hydrogen bonds with key amino acid residues such as GLU-235, while stabilizing the interaction through multiple non-covalent interactions including van der Waals forces, Pi-Sulfur interactions, carbon-hydrogen bonds, and Pi-Alkyl hydrophobic interactions; surface modeling revealed that AZ628 was completely embedded within the protein surface binding cavity with spatial conformation highly matched to the binding pocket. Refametinib also exhibited favorable binding affinity with ST3GAL4 protein (binding energy of -7.2 kcal/mol), primarily establishing specific interactions through conventional hydrogen bonds with amino acid residues such as ASN-149, with enhanced binding stability through multiple non-covalent bonds including van der Waals forces, Pi-Pi stacking interactions, carbon-hydrogen bonds, and Pi-Alkyl hydrophobic interactions; surface modeling showed Refametinib deeply buried within the hydrophobic cavity of the protein surface, with a binding mode consistent with the ligand’s chemical structural features. According to established molecular docking standards, binding energy < -5.0 kcal/mol indicates excellent binding affinity, and both ligands in this study exhibited binding energies far below this threshold, demonstrating strong and stable interactions with ST3GAL4 protein. These docking results suggest possible binding compatibility between ST3GAL4 and the candidate compounds AZ628 and Refametinib, providing preliminary structural support for the observed drug sensitivity associations and a basis for future experimental validation and drug optimization studies.

## Discussion

We investigated the risk of aging-related tumor deterioration and its associated immune microenvironment by integrating multi-omics data, identifying the RAM signature, which is linked to a reduced risk of aging-related tumor deterioration. Among the component features of RAM, multi-omics signals such as WNT5A have been implicated in the regulation of the tumor microenvironment and may reflect biologically relevant regulatory programs [62]. Although previous studies suggest that genetic, epigenetic, and transcriptional alterations may converge on shared aging-related pathways, the methylation-enriched and transcriptome-supported pattern of RAM should not be interpreted as evidence that these signals are biologically independent at the omics level. Instead, this pattern may reflect differences in how various molecular modalities capture age-associated variations.Specifically, DNA methylation and transcriptomic features more directly represent continuous aging-related regulatory state changes, while mutations and copy number variations (CNVs) are discrete genomic events influenced by tumor evolution and clonal selection, contributing less significantly to age-associated latent variation. This indicates that while functional convergence may exist at the pathway level, there may be unequal variance contributions at the latent factor level. Therefore, RAM should be viewed as a correlational, methylation-enriched, and transcriptome-supported multi-omics signature that captures a coordinated molecular state linked to aging-related phenotypes in melanoma. As previous studies have indicated, aging drives significant systemic alterations in both DNA methylation and RNA (mRNA) processing, fundamentally altering how genes are activated and silenced [63].

At the single-cell level, RAM was associated with distinct patterns of melanoma microenvironment organization. RAM+ cells were enriched for endothelial cells and fibroblasts, whereas RAM- cells were enriched for melanoma cells, proliferative melanoma cells, and myeloid cells. These findings suggest that RAM-associated transcriptional states are not randomly distributed across annotated cell types. Functional enrichment further showed that RAM+ cells were mainly linked to vascular-stromal remodeling and mitochondrial metabolic activity, whereas RAM- cells were associated with inflammatory activation, oncogenic signaling, hypoxia, epithelial-mesenchymal transition, and proliferative tumor programs. Together, these results suggest that decreased RAM may accompany a shift from a relatively homeostatic stromal-metabolic state toward a more aggressive, stress-adaptive, and tumor-permissive microenvironment.

Intercellular communication analysis further supported this interpretation. RAM- groups displayed stronger malignant-cell-centered communication, whereas RAM+ groups were characterized by more prominent stromal interactions, particularly involving endothelial cells and fibroblasts. Pathway-level analysis identified differential signaling activity in MIF, LAMININ, COLLAGEN, and TIGIT-related pathways, indicating that RAM status may be associated with distinct immune and structural communication programs in the tumor microenvironment. In particular, the selective enrichment of immune-inhibitory signaling in RAM- cell populations is consistent with an immunosuppressive niche that may be related to tumor progression and immune evasion. SCENIC analysis provided an additional layer of support by showing distinct transcriptional regulatory programs in RAM + and RAM- cells, with RAM+ cells enriched for factors such as ZFP42 and IRF8, and RAM- cells enriched for regulators including ILF2 and ISL1. These transcriptional differences suggest that RAM-associated cellular states are associated with distinct regulatory programs and may reflect divergent biological trajectories in melanoma.

Importantly, RAM-associated cell states were also linked to immunotherapy response and survival. In the immunotherapy cohorts, RAM+ fibroblasts and RAM- malignant cells showed differential abundance and communication patterns, and their estimated proportions correlated with clinical outcomes. These findings suggest that RAM-related cellular composition in the tumor microenvironment may be associated with response to immune checkpoint blockade. To further explore this possibility, we developed a machine learning-based predictive model using RAM cell marker genes. The model identified five core genes-GPR143, ST3GAL4, RAB38, GMPR, and FDFT1-that showed consistent importance across multiple feature-selection algorithms and demonstrated encouraging predictive performance in melanoma cohorts. These genes were also associated with immune infiltration patterns in SKCM, supporting their potential relevance to the tumor immune microenvironment. Together, these results suggest that RAM-associated markers may serve as candidate biomarkers for immunotherapy response, although their predictive value requires further validation in larger, well-annotated external cohorts.

We further examined the therapeutic relevance of the RAM signature by integrating GDSC data and molecular docking analyses. RAM-associated genes showed correlations with sensitivity to MAPK-pathway-targeted agents, including Dabrafenib, Trametinib, AZ628, and Refametinib. Among these genes, ST3GAL4 emerged as a potentially interesting candidate, given its association with immune infiltration, drug sensitivity, and favorable docking interactions with AZ628 and Refametinib. As a sialyltransferase previously implicated in melanoma progression and immune evasion, ST3GAL4 may represent a biologically relevant node connecting tumor glycosylation, immune escape, and therapeutic response. These results provide a rationale for further functional investigation of RAM-associated markers in melanoma.

This study has several important limitations that should be explicitly acknowledged. First, although our findings suggest associations between the RAM factor, cellular states, molecular markers, drug sensitivity, and clinical outcomes, they do not establish causal relationships or mechanistic links. Although covariate-adjusted analyses were performed where possible, residual confounding from unmeasured clinical, molecular, or treatment-related variables cannot be fully excluded. Second, because this study integrates multiple public cohorts generated across different platforms, sample-processing protocols, and patient populations, cohort heterogeneity and batch-related effects may influence the observed associations despite normalization and validation efforts. Third, the use of machine learning models may introduce risks of overfitting, particularly in settings with high-dimensional molecular features and limited sample sizes. Therefore, the predictive performance of RAM-associated models should be interpreted cautiously and requires further validation in independent prospective cohorts. Finally, molecular docking provides only theoretical binding predictions and cannot substitute for biochemical or cellular validation. Future studies should incorporate functional perturbation assays, in vivo models, longitudinal sampling, and prospective clinical validation to determine the mechanistic and translational significance of RAM and its associated markers.

## Supplementary Information

Below is the link to the electronic supplementary material.

Supplementary Material 1

Supplementary Material 2

Supplementary Material 3

## Data Availability

All data are publicly available. Transcriptome data, clinical data, somatic mutation data, copy number data, and methylation data were available at the Genomic Data Commons (https://gdc.cancer.gov/). Reverse phase protein array (RPPA) data was obtained from the cancer proteome atlas (TCPA) (https://tcpaportal.org/tcpa/index.html). GTEx data was obtained from The Genotype-Tissue Expression (GTEx) datasets (V8.0) (https://commonfund.nih.gov/GTEx/). The immunotherapy datasets were obtained Gene Expression Omnibus 41 (http://www.ncbi.nlm.nih.gov/geo), IMvigor210 study (http://research-pub.gene.com/IMvigor210CoreBiologies/) and SRA database (https://www.ncbi.nlm.nih.gov/bioproject). Single-cell RNA-sequencing data obtained from TISCH (http://tisch.comp-genomics.org/). Software and resources used for the analyses are described in each method section.
