# Supplementary material for: Single-cell multi-omics dissection of RevitalAge Markers uncovers age-dependent immunotherapy resistance and druggable targets in melanoma
Source: Biol Direct. 2026 Jun 16;21:128. doi: 10.1186/s13062-026-00860-x (PMC13383403; doi:10.1186/s13062-026-00860-x)
Supplement: Supplementary file 1 — Supplementary Material 1 [file 13062_2026_860_MOESM1_ESM.docx]

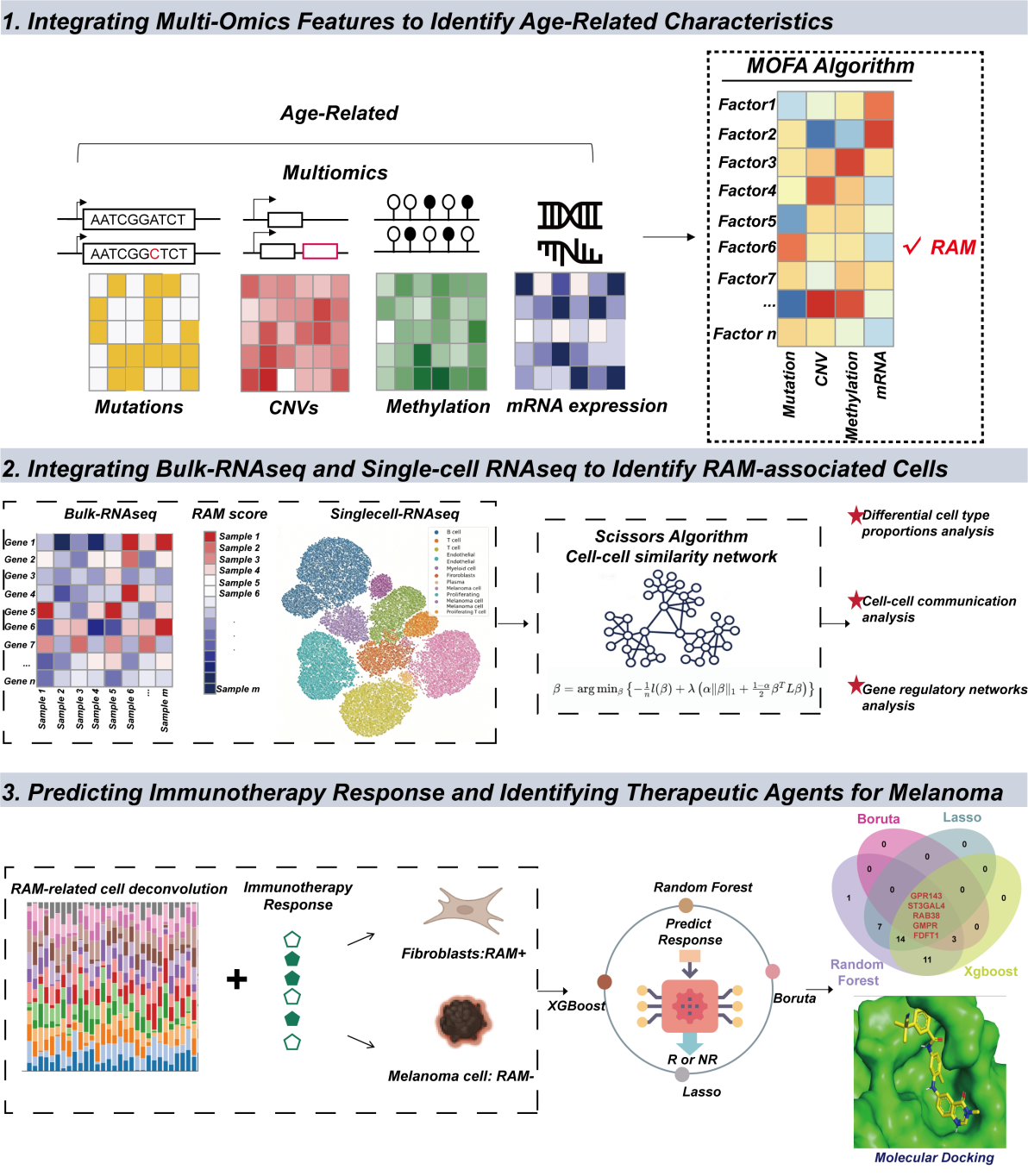


**Figure S1. Multivariable analysis of RAM-related clinical associations in melanoma.**


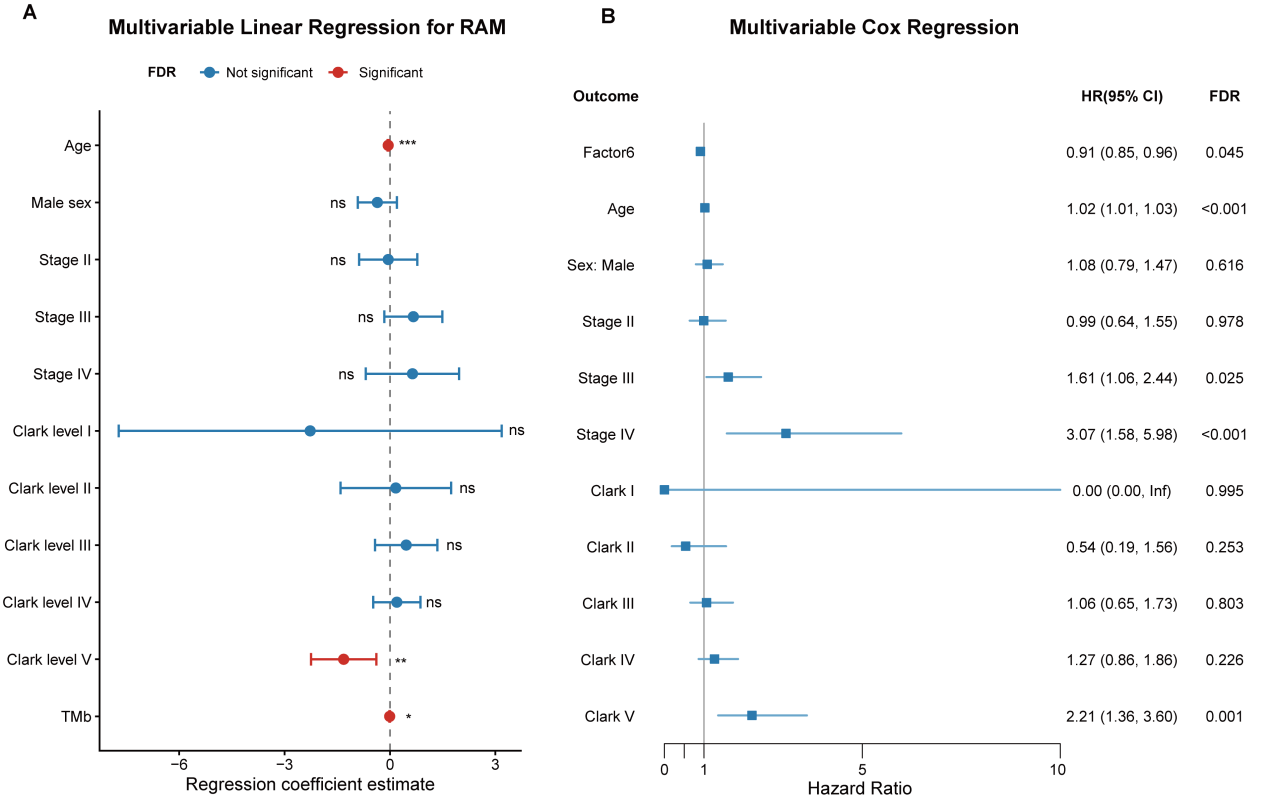


**Figure S2. Multivariable analysis of RAM-related clinical associations in melanoma.**(A) Multivariable linear regression assessing associations between RAM scores and clinical variables, including age, sex, stage, Clark level, and TMB. Points indicate regression coefficients with 95% confidence intervals.
(B) Multivariable Cox regression evaluating the association between RAM/Factor 6 and overall survival after adjustment for clinical covariates. Points indicate hazard ratios with 95% confidence intervals; FDR values are shown on the right.


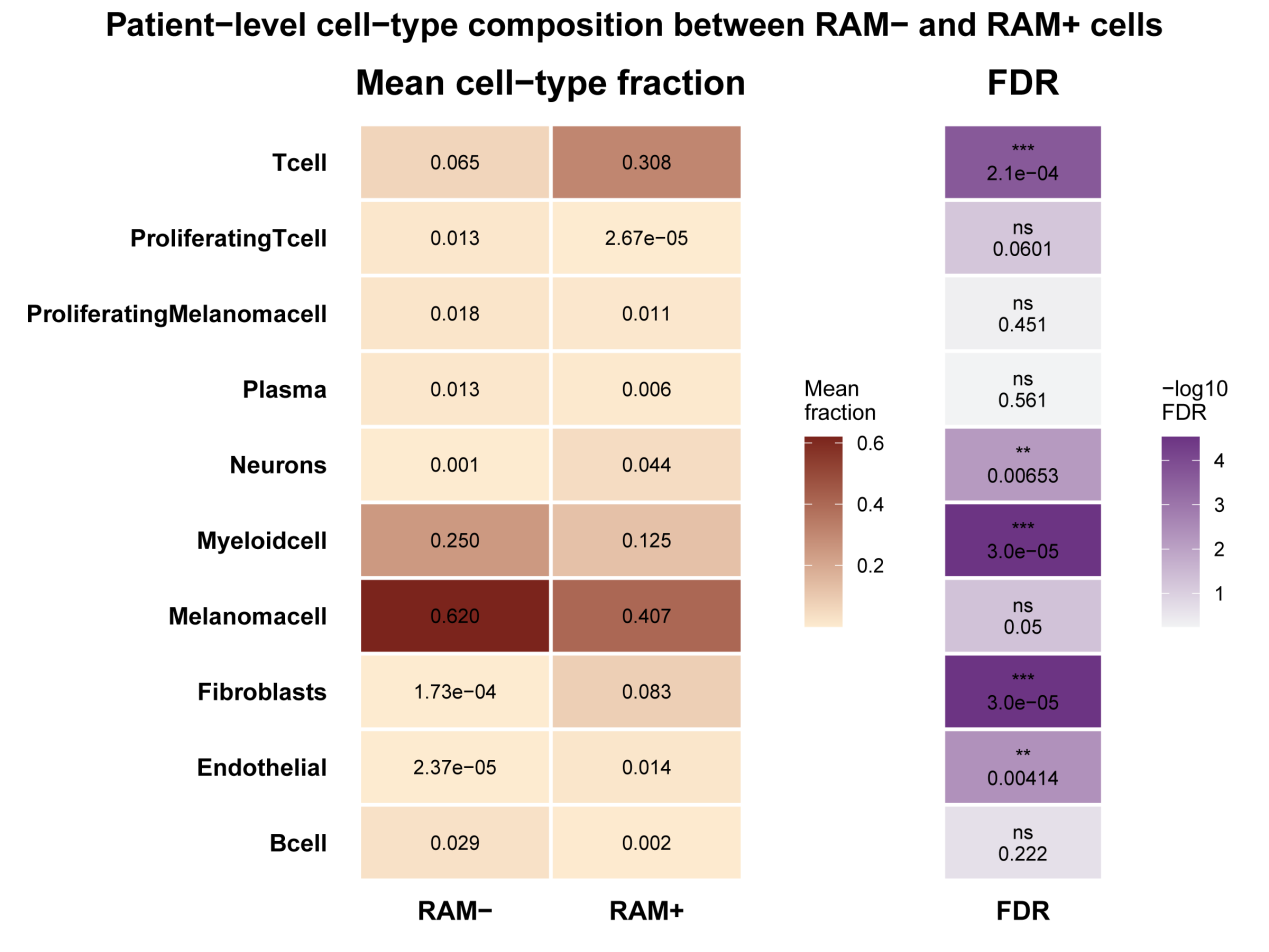


**Figure S3. Cell-type composition of RAM− and RAM+ groups.** Heatmap showing patient-level mean fractions of annotated cell types in RAM− and RAM+ groups.


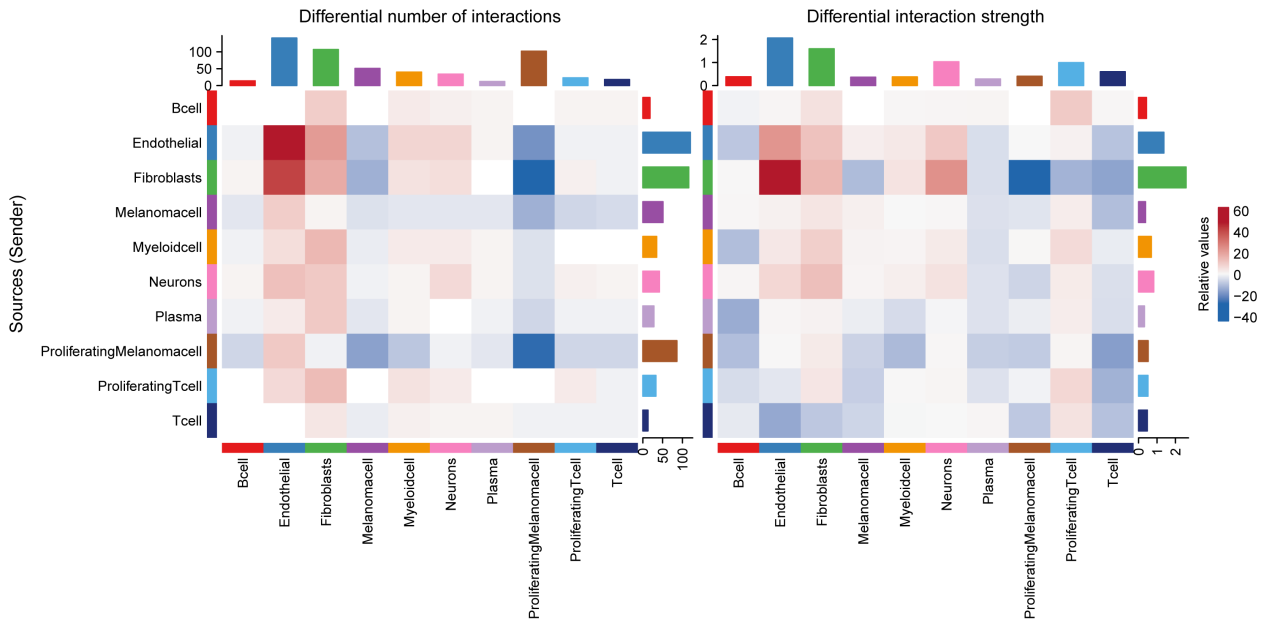


**Figure S4. Differential cell–cell communication patterns between RAM− and RAM+ groups.**


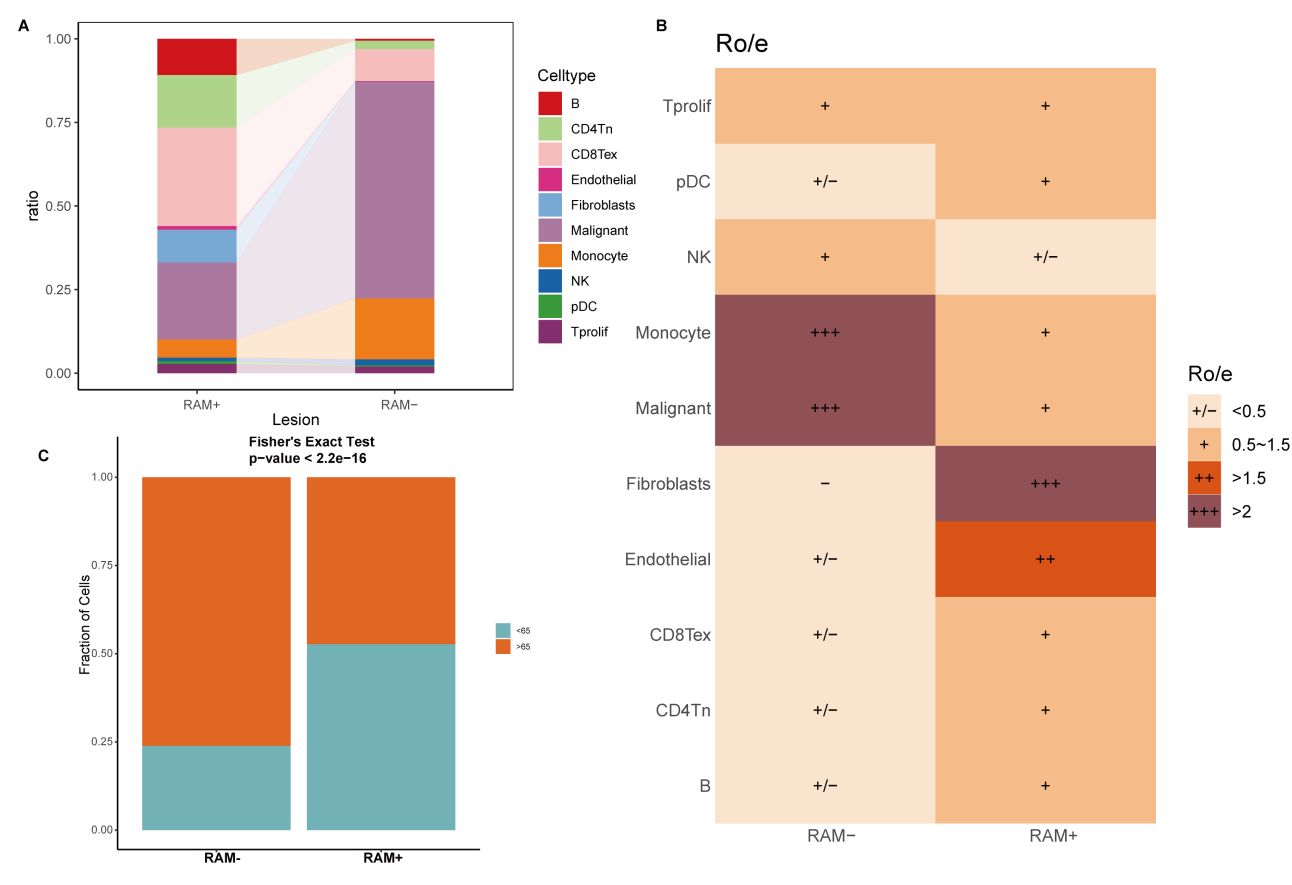


**Figure S5. Aging-associated RAM status defines distinct tumor microenvironment landscapes in melanoma.**(A) Cell type composition in RAM- and RAM+ samples. (B) Heatmap showing cell type enrichment patterns (Role score) with statistical significance indicated by asterisks (* P < 0.05, ** P < 0.01, *** P < 0.001). (C) Lesion-specific cell distribution comparison (Fisher's exact test, P < 2.2e-16). RAM- samples demonstrate significant enrichment of monocytes and stromal components, while RAM+ samples show increased malignant cell proportions.


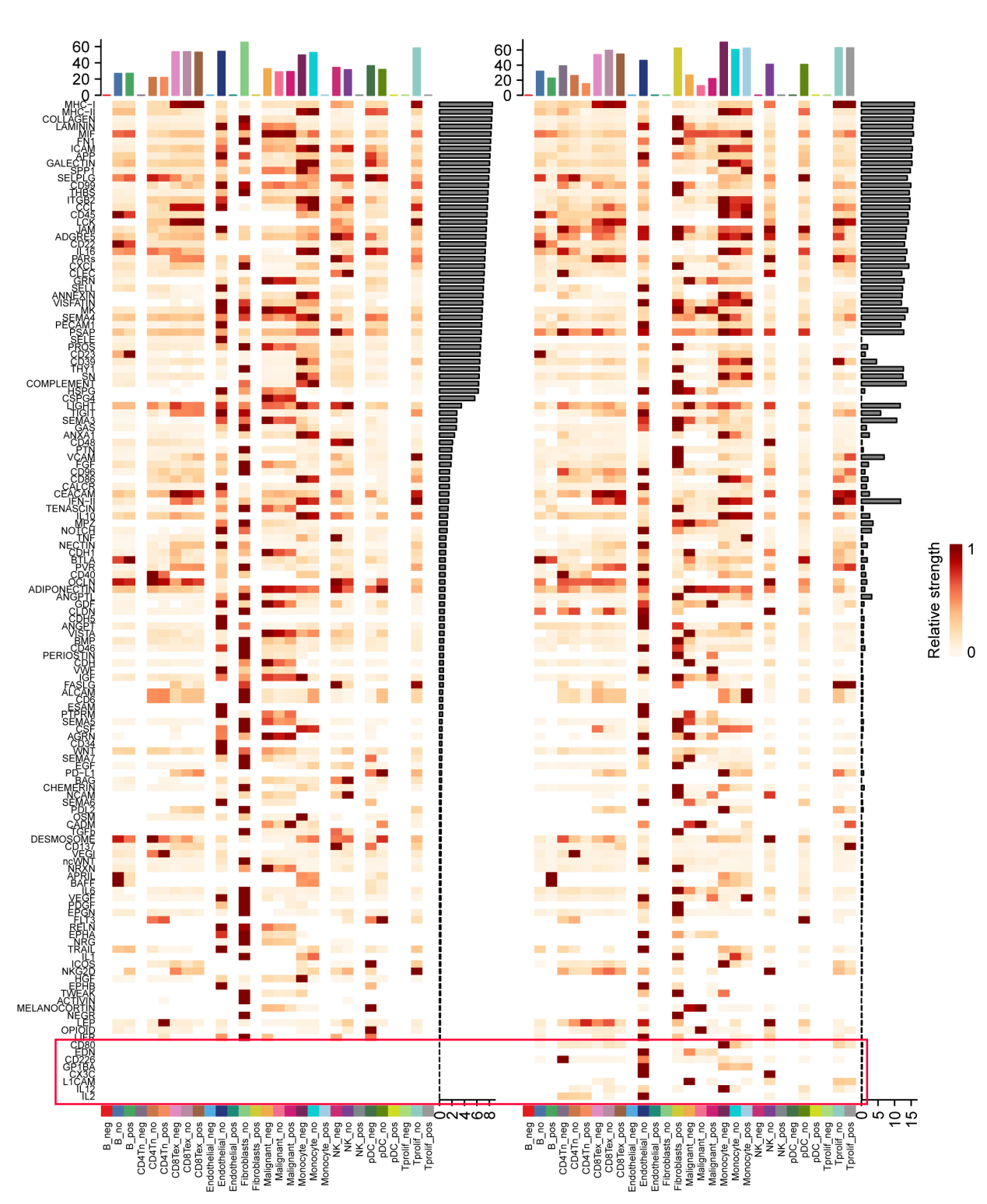


**Figure S6.:Cell-cell communication analysis reveals differential signaling patterns between RAM+ and RAM- melanoma cells in immunotherapy-treated versus untreated samples.**


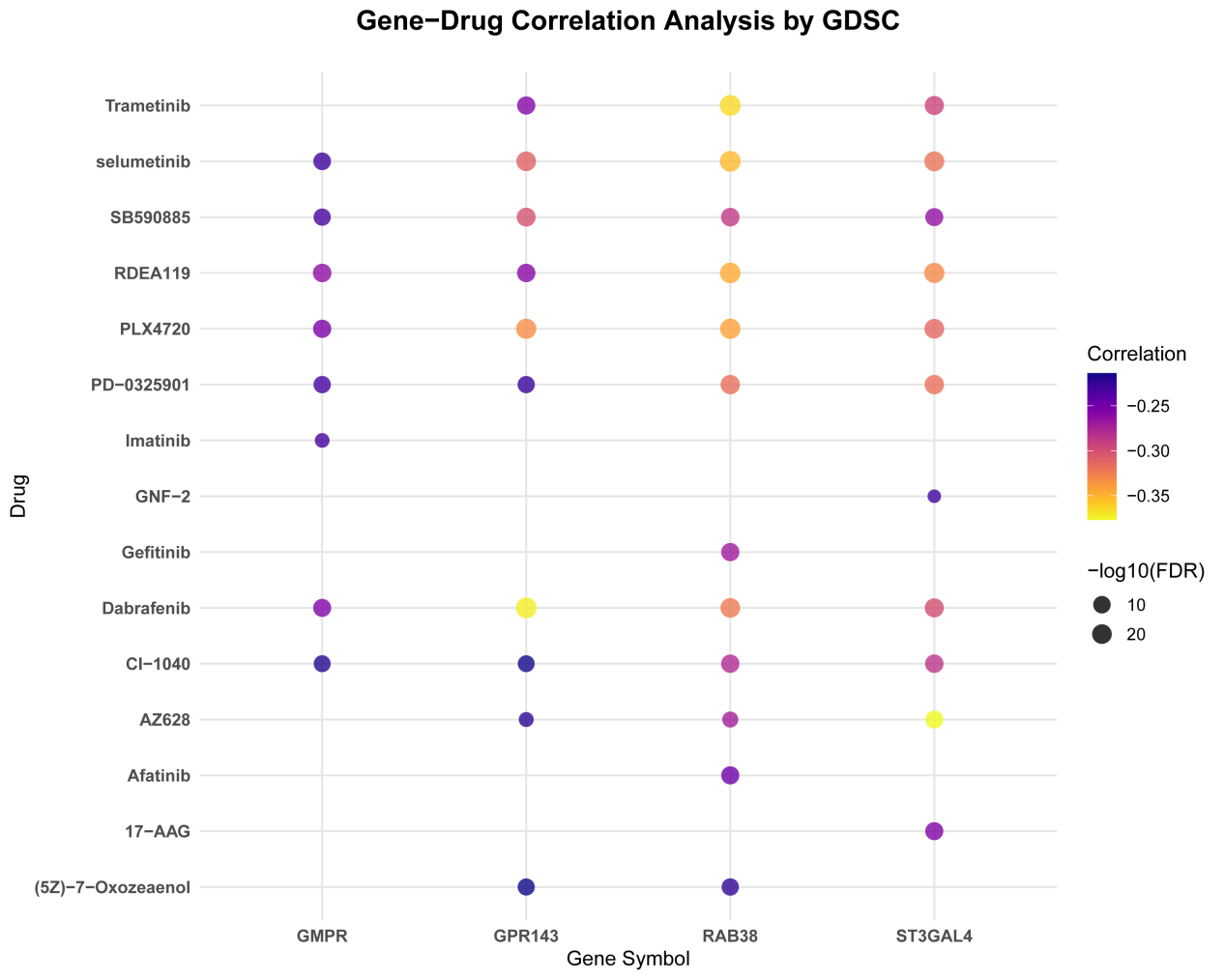


**Figure S7: Gene-Drug Correlation Analysis identifies RAM signature genes as predictors of therapeutic response in melanoma.**


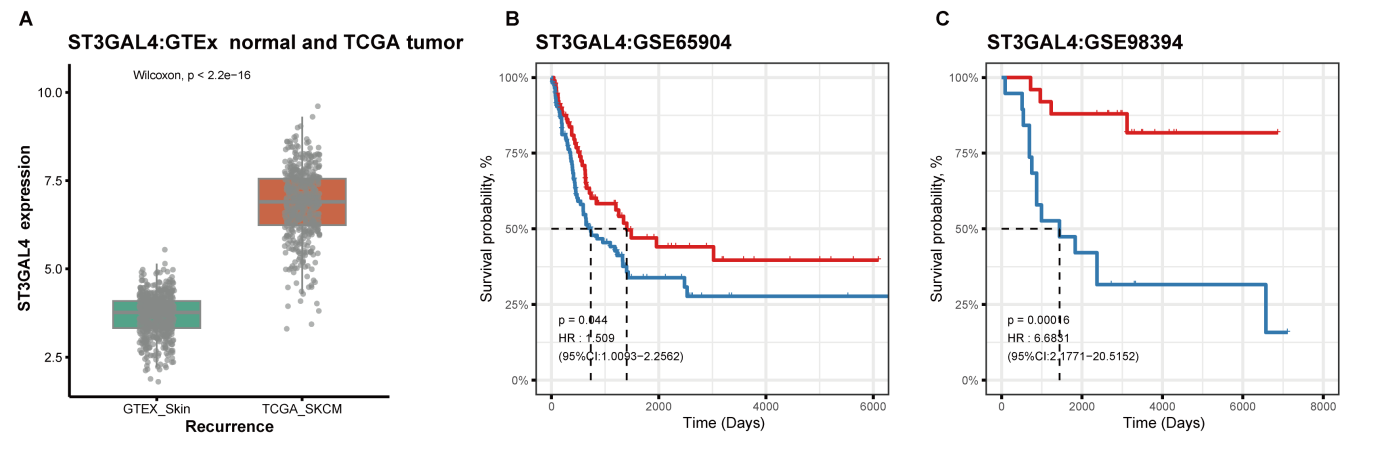


**Figure S8. ST3GAL4 expression and prognostic relevance in melanoma.**

A, ST3GAL4 expression was compared between GTEx normal skin and TCGA-SKCM tumor samples. Statistical significance was assessed using the Wilcoxon test. B–C, Kaplan–Meier survival curves showing the association between ST3GAL4 expression and patient survival in the GSE65904 and GSE98394 melanoma cohorts. Patients were stratified into high- and low-expression groups according to ST3GAL4 expression. P values were calculated using the log-rank test. Red and blue curves indicate high- and low-ST3GAL4 expression groups, respectively..
